# Advanced Extraction of Lipids with DHA from *Isochrysis galbana* with Enzymatic Pre-Treatment Combined with Pressurized Liquids and Ultrasound Assisted Extractions

**DOI:** 10.3390/molecules25143310

**Published:** 2020-07-21

**Authors:** María Señoráns, Natalia Castejón, Francisco Javier Señoráns

**Affiliations:** Healthy-Lipids Group, Sección Departamental de Ciencias de la Alimentación, Faculty of Sciences, Universidad Autónoma de Madrid, 28049 Madrid, Spain; mariasmab@gmail.com (M.S.); natalia.castejonc@gmail.com (N.C.)

**Keywords:** omega-3 PUFA, microalgae, advanced extraction techniques, pressurized liquid extraction (PLE), enzymatic pre-treatment, *Isochrysis galbana*, UAE, polar lipids

## Abstract

Microalgal biomass is a sustainable and valuable source of lipids with omega-3 fatty acids. The efficient extraction of lipids from microalgae requires fast and alternative extraction methods, frequently combined with biomass pre-treatment by different procedures. In this work, Pressurized liquid extraction (PLE) was optimized and compared with traditional lipid extraction methods, Folch and Bligh and Dyer, and with a new Ultrasound Assisted Extraction (UAE) method for lipids from microalgae *Isochrysis galbana*. To further optimize PLE and UAE, enzymatic pre-treatment of microalga *Isochrysis galbana* was studied with commercial enzymes Viscozyme and Celluclast. No significant differences were found for lipid yields among different extraction techniques used. However, advanced extraction techniques with or without pre-treatment are a green, fast, and toxic solvent free alternative to traditional techniques. Lipid composition of *Isochrysis* was determined by HPLC-ELSD and included neutral and polar lipids, showing that each fraction comprised different contents in omega-3 polyunsaturated fatty acids (PUFA). The highest polar lipids content was achieved with UAE (50 °C and 15 min) and PLE (100 °C) techniques. Moreover, the highest omega-3 PUFA (33.2%), eicosapentaenoic acid (EPA) (3.3%) and docosahexaenoic acid (DHA) (12.0%) contents were achieved with the advanced technique UAE, showing the optimized method as a practical alternative to produce valuable lipids for food and nutraceutical applications.

## 1. Introduction

Microalgae are potential sources of novel and biologically active compounds with numerous applications in pharmaceutical, cosmetic and food industries [1]. In recent years, microalgae have attracted increasing interests due to their diverse biochemical composition. Marine microalgae have a high content of proteins and lipids, carotenoids, phenolic compounds and long-chain polyunsaturated fatty acids (LC-PUFAs), among other bioactive compounds [2,3].

Lipids present in microalgae can be classified into neutral lipids (acylglycerols, sterols, waxes, free fatty acids (FFA)) and polar lipids (glycolipids and phospholipids). Lipid composition and content mainly depend on the microalgae species and time and conditions of cultivation. [4,5]. Neutral lipids, mainly triacylglycerols (TAGs), are accumulated as lipid droplets in the cytosol, while polar lipids are mostly found in microalgae membranes [6]. Polar lipids can accumulate more long-chain fatty acids and omega-3 LC-PUFA (n-3 LC-PUFA) [7].

The n-3 LC-PUFAs, such as eicosapentaenoic acid (EPA, C20:5n-3) and docosahexaenoic acid (DHA, C22:6n-3), have great benefits for human health. The World Health Organization (WHO) indicates that n-3 LC-PUFAs reduce the risk of cardiovascular diseases with a recommended intake of 0.250 g per day for adult men and women (no pregnant or lactating women) of EPA and DHA [8]. N-3 LC-PUFAs are also important in the growth and development of infant brain, the regulation of blood pressure, renal function, blood coagulation and inflammatory and immunological reactions [9,10].

The main natural dietary source of EPA and DHA is fish; however, world fisheries cannot meet the global demand for n-3 fatty acids. There is therefore a need to find novel and sustainable sources of these bioactive compounds. In this sense, microalgae are considering potential sustainable sources of n-3 LC-PUFAs [11,12] due to their advantages over terrestrial crops such as higher growth rate, higher biomass production and smaller cultivation area [13].

*Isochrysis galbana* microalga can synthesize EPA and DHA [14] and is also interesting for its content in vitamins, polysaccharides, sterols and carotenoids (mainly chlorophylls a and c, fucoxanthin and diadinoxanthin); therefore, it is a valuable source for human and animal nutrition. In addition, it can grow in extreme environmental conditions, it has a high growth rate and high productivity [15].

Traditional extraction methods commonly used for the extraction of microalgae lipids are based on organic solvent systems, implying a high volume of toxic solvents. Some of the methods frequently used are Bligh and Dyer [16] and Folch methods [17], where compounds are extracted with a solvent mixture of chloroform: methanol. Due to the lipid composition of microalgae, mixtures of polar and non-polar solvents are needed to achieve effective extractions because these solvents allow the interaction with neutral and polar lipids [18]. However, long extraction times, large quantities of expensive and toxic solvents, limited selectivity and contamination are some of the problems presented by these classical methods [19,20]. Some solvents such as benzene, cyclohexane, acetone and chloroform are effective in lipid extractions from microalgae because these solvents degrade the cell wall of microalgae and they have high solubility with lipids [21], but some of these solvents are highly hazardous and must be avoided.

To avoid harmful effects for the environment and human health, new sustainable methods based on the principles of green chemistry [22,23] are being developed using advanced extraction techniques such as ultrasound assisted extraction (UAE) and pressurized liquids extraction (PLE). These methods avoid large amounts of toxic solvents and are fast and efficient techniques. These techniques are more economical, environmentally friendly and quicker than traditional techniques [24,25,26,27].

On the other hand, one of the most critical points in the extraction of bioactives from microalgae is the selection of an appropriate extraction technique, due to the presence of a dense and firm microalgae cell wall. For that reason, the microalgae cell wall must be properly disrupted to efficiently recover intracellular bioactive compounds [5]. The microalgae cell wall is generally composed by polysaccharides such as cellulose, uronic acid, mannose, xylan, algaenan, glycoproteins and minerals [28]. Among the cell wall disruption methods include physical methods such as UAE, bead-beating, microwaves, homogenization, enzymatic methods and chemical methods [29,30]. In this regard, PLE and UAE could be the key to develop new and environmentally friendly extraction methods from microalgae, due to its demonstrated effective acting in cell membranes and cost competitive results [31,32].

The use of enzymes facilitates the hydrolysis of microalgae cell walls, and if the enzymes are combined with other physical disruption methods, fast extraction and highest extraction yields are obtained [21,33]. Previous studies of the research group demonstrated that it was possible to enrich and fractionate neutral and polar lipids using enzyme pre-treatment and PLE from microalgae [34]. Other authors have also reported the use of enzymes such as lysozyme and cellulase to break the cells of wet microalgae achieving higher lipid extraction yields [29].

Thus, the main objective of this study was to assess the use of advanced extraction techniques such as pressurized liquids and ultrasounds in combination with enzymatic pre-treatments, to efficiently extract the lipids from the microalgal biomass of *Isochrysis galbana*, and to fractionate neutral glycerides and polar glycolipids with different omega-3 PUFA composition. Additionally, lipid classes and fatty acid composition of extracted fractions using different extraction conditions and techniques were analyzed by HPLC-ELSD and GC-MS to evaluate the total lipid recovery and the different fractionation of polar and non-polar lipids of microalga extracts, to take full advantage of their omega-3 PUFA content.

## 2. Results and Discussion

### 2.1. Lipid Extraction from Isochrysis galbana Biomass Using Traditional Techniques

To determine the total lipid content, traditional extraction techniques were applied to the microalgal biomass of *Isochrysis galbana*. Bligh and Dyer and Folch methods based on chloroform:methanol solvent systems have been widely used as standard methods for lipid recovery from seaweeds and microalgae. In this work, the Folch method reached an extraction yield of 13.8% ± 1.1, while using the Bligh and Dyer method a lipid yield of 18.4% ± 0.1 was achieved. The use of a large proportion of methanol in the Bligh and Dyer method could be the reason for the higher extraction yield. Other authors have also reported a higher extraction yield using the Bligh and Dyer method due to the presence of polar compounds in the extracted mixture, such as carbohydrates and pigments [35]. For that reason, in this study, the Folch method was selected as the standard traditional technique and it will be used to compare further results using advanced extraction methods. Some authors even reported that the use of organic solvents such as chloroform have the ability to weaken the rigid cell walls of microalgae [31,36], the main mechanism of these traditional extraction methods consist in penetration and membrane diffusion. Thus, to achieve a better extraction yield, other techniques implying the disruption of microalgae cell walls were investigated (see Section 2.3).

### 2.2. Lipid Extraction from Isochrysis galbana Biomass Using Advanced Extraction Techniques

#### 2.2.1. Ultrasound Assisted Extraction

**Figure 1:**
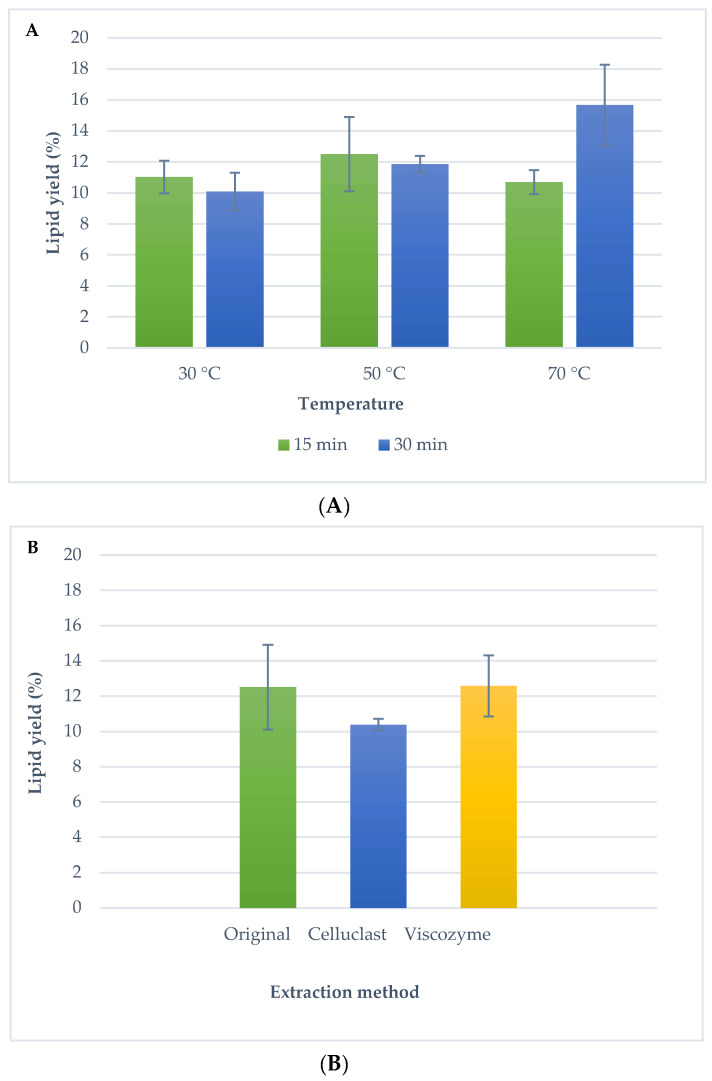
(**A**) Microalga lipid yield obtained by Ultrasound Assisted Extraction (UAE) using different temperatures (30 °C, 50 °C and 70 °C) and times (15 min and 30 min); (**B**) Microalga lipid yield obtained by UAE (50 °C 15 min) using different enzymatic pre-treatment (Celluclast and Viscozyme). Error bars denote the standard deviation of two independent determinations.

As an alternative method to the traditional techniques, lipid extraction from *Isochrysis galbana* was evaluated using UAE. Microalgal lipids were extracted using ultrasounds and a mixture of hexane:isopropanol (2:1). It should be highlighted that a mixture of polar and non-polar solvents is necessary to recover the broad spectrum of microalgae lipids; this solvent mixture was successfully used by a research group in a previous study [34]. Figure 1a shows extraction yields reached by UAE using different temperatures (30 °C, 50 °C and 70 °C) and extraction times (15 min and 30 min). Although the highest lipid yield was reached at a temperature of 70 °C and 30 min (15.7% ± 2.6), these conditions were not chosen as optimal because the temperature was considered high for the thermolabile microalgal compounds, in case carotenoid components are extracted together with omega-3 fatty acids [18]. Using the mid temperature investigated (50 °C), a lipid yield of 12.5% ± 2.4 and 11.9 ± 0.5 were achieved using 15 min and 30 min, respectively (no significant differences at 5% level). Thus, in order to prevent oxidation processes, 50 °C and 15 min was selected as optimal conditions for UAE. Comparing with traditional techniques, a similar lipid recovery to Folch was obtained, but UAE is a greener extraction alternative using non-toxic solvents, in a fast and a simple method.

#### 2.2.2. Pressurize Liquid Extraction

**Figure 2:**
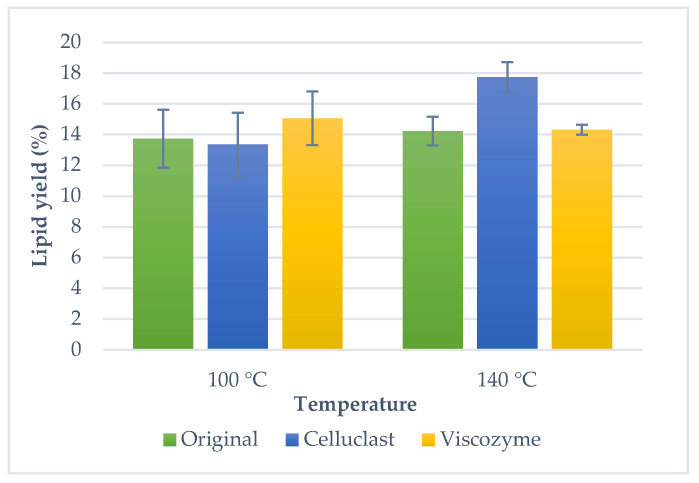
Microalga lipid yield obtained by pressurized liquids extraction (PLE) using different temperatures (100 °C and 140 °C) with and without enzymatic pre-treatments. Error bars denote the standard deviation of two independent determinations.

Another alternative technique to traditional methods is pressurized liquids extraction, due to the high pressures and temperatures used, higher extraction yields are expected. Microalgal lipids were extracted with PLE using the same solvent mixture than UAE, hexane:isopropanol (2:1). Moreover, different temperatures were investigated (100 °C and 140 °C); extraction yields are shown in Figure 2. Lipid yields were 13.7% ± 1.9 and 14.2% ± 0.9 for 100 °C and 140 °C, respectively. The increase of temperature did not imply a highest extraction yield for the microalgal biomass using PLE (no significant differences at 5% level). Therefore, the optimal extraction conditions for the PLE were selected at 100 °C. Despite having an extraction yield similar to Folch method (no significant differences at 5% level), PLE technique is a good alternative to the traditional method since it involves rapid and automated extractions and less volume of toxic solvents.

Comparing UAE and PLE, no significant differences were found in the lipid yield for the optimal extraction conditions. Thus, both advanced extraction techniques can be used as alternatives to traditional methods to extract microalgae lipids from *Isochrysis galbana*, achieving faster, more economical and less toxic extraction methods.

### 2.3. Lipid Extraction from Enzymatic Pre-Treated Microalgal Biomass Using Advanced Extraction Techniques

In a further step, the effect of enzymatic pre-treatment on the lipid recovery from *Isochrysis galbana* using advanced extraction techniques was investigated. Two commercial enzymes were used, Celluclast and a mixture of carbohydrases including arabinase, cellulase, beta-glucanase, hemicellulase and xylanase (Viscozyme), with the final objective of disrupting the cell wall of the microalgae, and therefore, improving the lipid extraction yield. Commercial enzymes have shown to effectively improve the extraction yield of different enzymatic extracts from microalgae [29,33].

First, to optimize the enzymatic pre-treatment, different incubation times (2, 4, 8 and 24 h) followed by the extraction using ultrasounds at the optimal conditions (50 °C and 15 min) for Celluclast and Viscozyme were carried out. Among the different incubation times, no significant differences were found for any of the tested enzymes (data not shown). Thus, an incubation time of 2 h was selected to carry out further extraction experiments.

Figure 1b shows the lipid extraction of the pre-treated biomass using ultrasounds. In general, similar lipid yield was reached with UAE and enzymatic treatment compared to UAE without pre-treatment. A lipid yield of 12.5% ± 2.4 was achieved for the UAE technique without enzymatic pre-treatment, 10.4% ± 0.3 for Celluclast and UAE and 12.6% ± 1.73 for Viscozyme and UAE (no significant differences at 5% level).

Similar behavior was found for the lipid extraction of the pre-treated biomass using pressurized liquids. An enzyme treatment (Viscozyme and Celluclast) was performed previous to PLE extraction with different temperatures (100 °C and 140 °C) (Figure 2). At the lowest temperature, 13.7% ± 1.9 lipid yield was achieved with PLE, 13.4% ± 2.1 for Celluclast treatment and 15.1% ± 1.75 for Viscozyme treatment, however, they did not show significant differences at 5% level. Meanwhile, when the temperature increased, 14.2% ± 0.9 lipid yield was achieved with PLE without pre-treatment and the highest lipid yield was obtained with Celluclast and PLE (17.7% ± 1.0) (no significant differences at 5% level were found). Finally, a Viscozyme pre-treatment did not mean an increase in extraction yield (14.3% ± 0.3) (no significant differences at 5% level), nevertheless, different classes of lipids were found (see the fractionation of lipid classes in Section 2.4). Therefore, optimal extraction conditions for PLE at the lowest temperature (100 °C) were chosen. In conclusion, with enzymatic pre-treatment, membranes and other cellular structures were broken, different lipids were released and subsequent extraction by UAE and PLE was facilitated, thus, it is important to characterize the composition of the extracts achieved to adequately assess the possible advantages of the application of enzymes.

To determine the most suitable technique for omega-3 fatty acids extraction from microalgae contained in complex lipids (including glycolipids), beyond the differences in total extraction yields, it is essential to characterize the extracts obtained. Previous studies have shown that the composition of the extracts achieved is very different depending on the conditions and extraction technique used, both in relative composition and distribution of the types of lipids, and in their omega-3 content, thus, this characterization is crucial for choosing the optimal extraction conditions.

### 2.4. Analysis of Isochrysis galbana Lipid Classes by HPLC-ELSD

**Figure 3:**
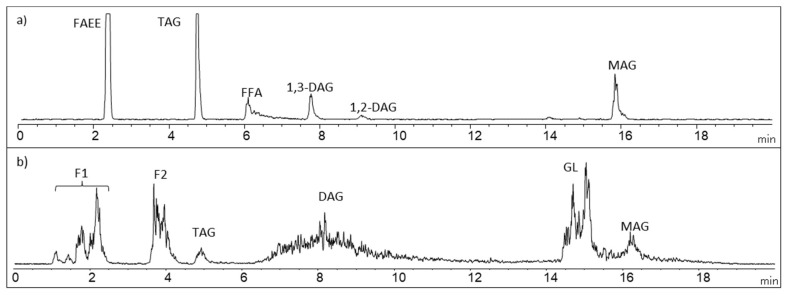
Example of lipid profile of standard mixture (**a**), extracted *Isochrysis galbana* by PLE at 100 °C (**b**), determined by HPLC-ELSD. Fatty acids ethyl ester (FAEE); Triacylglycerol (TAG); Free fatty acid (FFA); 1,3-diacylglycerol (1,3-DAG); 1,2-diacylglycerol (1,2-DAG); Monoacylglycerol (MAG); Fraction 1 (F1) and Fraction 2 (F2); Glycolipid (GL).

Lipid classes of *Isochrysis galbana* were analyzed by HPLC with Evaporative Light Scattering Detector (ELSD) in order to evaluate the lipid composition obtained with different techniques. The lipid profiles of all microalgal extracts were analyzed. Figure 3 shows an example of an HPLC-ELSD chromatogram of the lipid profile of *Isochrysis galbana.* The analysis by HPLC allowed to identify in *Isochrysis galbana* a first fraction (F1) that contained squalene, esterified cholesterol and waxes and a second fraction (F2) that contained possible alquenones; these compounds were shown in some studies [37,38]. Triglycerides (TAG), free fatty acids (FFA), diglycerides (DAG), glycolipids (GL) and monoglycerides (MAG) were also identified. Other authors also identified a similar lipid profile but reporting different lipid percentages [39,40].

**Figure 4:**
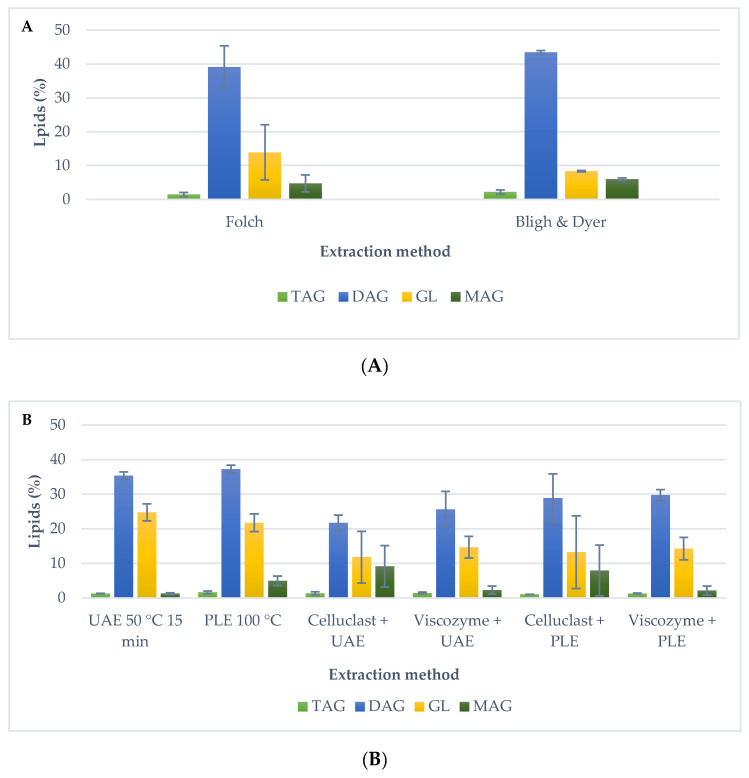
TAG, DAG, GL and MAG content obtained by different techniques. (**A**) Traditional methods Folch and Bligh and Dyer and (**B**) advanced extraction techniques at the optimal extraction condition and enzyme pre-treatment and not.

Figure 4 shows the relative content of TAG, DAG, MAG and GL in the extracts, attained with the different extraction methods with and without the enzymatic pre-treatment.

Generally, the TAG content was low, although there were significant differences at 5% level between the different methods. Highest content of TAG was achieved with Bligh and Dyer method (2.2% ± 0.6) followed by PLE method without pre-treatment (1.6% ± 0.4). The lowest TAG content was obtained with Celluclast pre-treatment and PLE (1.0% ± 0.0). The pre-treated samples did not improve the TAG content in the case of using UAE technique with Viscozyme and Celluclast (UAE 1.3% ± 0.1, Celluclast + UAE 1.3% ± 0.4 and Viscozyme 1.5% ± 0.2) (no significant differences at 5% level). Moreover, a similar TAG content was achieved using the PLE technique without enzymatic pre-treatment and PLE with Celluclast and Viscozyme (PLE 1.6% ± 0.4, Celluclast and PLE 1.0% ± 0.0, Viscozyme and PLE 1.3% ± 0.1) (no significant differences at 5% level), although significant differences at 5% level were found between Celluclast and Viscozyme enzymes.

Regarding the DAGs, the highest content was found for the extracts obtained with traditional techniques (39.1% ± 6.2 to 43.5% ± 0.5). When lipids were extracted with UAE (35.4% ± 1.1) and PLE (37.3% ± 1.2), DAG content decreased by 5% (significant differences at 5% level). Nevertheless, no differences were reached between the extraction using UAE and PLE (no significant differences at 5% level). A different behavior was found when an enzymatic pre-treatment was carried out. A lowest DAG content was reached for Celluclast and UAE extraction (21.7% ± 2.2) (significant differences at 5% level), and it was similar for Viscozyme and UAE (25.6% ± 5.1) (no significant differences at 5% level). Moreover, in the case of the enzymatic treatment and PLE, DAG content was similar between Celluclast and PLE (28.8% ± 7.0) and PLE without enzymatic treatment (no significant differences at 5% level) and a lowest DAG contained was achieved for Viscozyme and PLE (29.8% ± 1.6) (significant differences at 5% level) regarding the sample extracted without pre-treatment and PLE. In addition, no significant difference al 5% level were found between the two enzymes tested.

The GL content was higher in the extracts using advanced techniques, UAE and PLE, without enzymatic pre-treatment, 21.7% ± 3.0 and 24.8% ± 2.4, respectively. GL content was similar for Celluclast and UAE (11.7% ± 7.4), for Viscozyme and UAE (14.6% ± 3.1), for Celluclast and PLE (13.2% ± 10.5) and for Viscozyme and PLE (14.3% ± 3.2) (no significant differences at 5% level). On the other hand, the lowest GL value was achieved with traditional methods (13.9% ± 8.1 to 8.3 ± 0.2) (significant differences at 5% level). This result could be explained due to the high polar lipids content found in membranes and cell walls of microalgae. Traditional extraction methods did not produce the disruption of the cell wall and, as a consequence, polar lipids are not extracted with these techniques.

An interesting result of this study was the FFA content identified in the different extracts. In samples pre-treated and extracted with advanced techniques, FFA content was identified (27–37%) (no significant differences at 5% level). In contrast, in samples without pre-treatment and in traditional methods, FFA was not identified. As a result, this FFA content explains the lower content in DAG, MAG and GL of enzymatic pre-treatment extracts.

On the other hand, MAG content was generally low in all the produced extracts. Microalgal extracts obtained with Celluclast and UAE (9.2% ± 6.0) and Celluclast and PLE (7.9% ± 7.3) showed the highest MAG percentages, followed by traditional methods (6.0% ± 0.4 with Bligh and Dyer and 4.7 ± 2.5 with Folch), whereas the lowest MAG content was found in UAE extracts (1.3% ± 0.3). The enzymatic pre-treatment using Celluclast and Viscozyme improved the MAG extraction with UAE (significant differences at 5% level), in the case of PLE (5.0% ± 1.4), MAG content was not improved with Celluclast (7.9% ± 7.3) and with Viscozyme (2.1% ± 1.3) (no significant differences at 5% level).

To better understand the fractionation process, the content of neutral lipids (the sum of TAG, DAG, MAG) was compared with the identified polar lipids, as glycolipids. In general, a highest content of neutral lipids was found for all the extracts. Extracts reached using advanced extraction techniques without pre-treatment were characterized by a higher glycolipid content, 21.7% to 24.8% than the rest of the techniques. The lipid species content achieved with the traditional Folch technique was 45.3% of neutral lipids and 13.9% of polar lipids, while with Bligh and Dyer, results were comparable to the Folch method, since 51.7% of neutral lipids and 8.3% of polar lipid content was obtained.

In summary, neutral and polar lipids of *Isochrysis galbana* can be fractionated using advanced extraction techniques, with or without using an enzymatic pre-treatment, depending on the lipid species to be concentrated.

### 2.5. Determination of Fatty Acids in Isochrysis galbana Extracts

**Figure 5:**
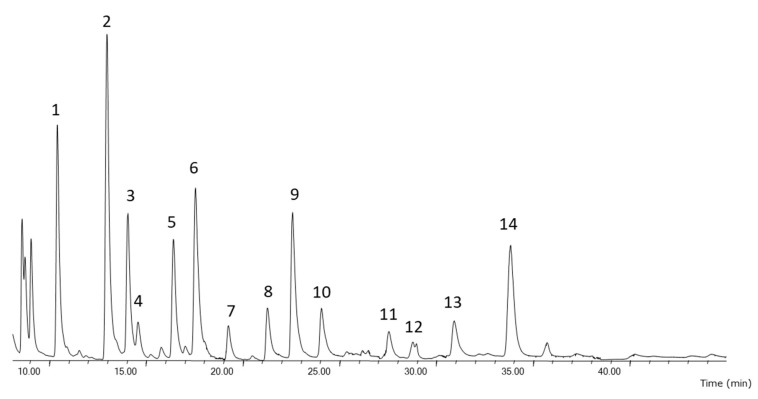
Fatty acid profile of microalgal lipid extracted with Folch traditional method determined by GC-MS. 1. C14: 0, 2. C16: 0, 3. C16: 1, 4. C: 17: 0, 5. C18: 0, 6. C18: 1, 7. C18: 2 n-6, 8. C18: 3 n-3, 9. C18: 4 n-3, 10. C20:4 n-6., 11. C20: 5 n-3, 12. Not identified (N.i.), 13. N.i., 14. C22: 6 n-3.

Fatty acid content of all microalgal lipids extracts was analyzed by Gas Chromatography-Mass Spectrometry (GC-MS) in order to evaluate the fatty acid composition reached with different techniques. In general, fatty acids identified (shown in Figure 5) were myristic acid (14:0), palmitic acid (16:0), palmitoleic acid (16:1 cis-9), oleic acid (18:1 cis-9) and stearidonic acid (18:4 cis-6,9,12,15), margaric acid (17:0), stearic acid (18:0), linoleic acid (18:2 cis-cis-9,12), α-linolenic acid (18:3 cis-9,12,15), arachidonic acid (20:4 cis-5,8,11,14), EPA (20:5 cis-5,8,11,14,17) and DHA (22:6 cis-4,7,10,13,16,19).

Table 1 shows the fatty acid composition (as a percentage of total fatty acids) of the extracts obtained at optimal extraction conditions from *Isochrysis galbana* comparing extraction techniques, solvents and the use of the enzymatic pre-treatment.

Comparing the saturated fatty acids (SFA) content between the extraction techniques, the highest SFA content was achieved with the traditional methods (44.1%) and the lowest SFA content was found in the advanced extraction techniques without pre-treatment (31.4%). A similar myristic acid and margaric acid content was found. Nevertheless, a higher palmitic acid content was achieved from traditional techniques than the other techniques. An interesting result was the stearic acid content; this fatty acid was not identified in samples extracted with PLE and UAE. In contrast, it was found in traditional techniques and pre-treatment extractions (2.3% to 8.7%).

Regarding the monounsaturated fatty acids (MUFA) content, a low percentage of these fatty acids were identified in all extracts (20.2% to 24.6%); however, differences were found between the traditional methods and the other extractions methods. The technique that rendered the lowest MUFA content was the Folch method (20.2%) and the highest MUFA content was UAE (24.6%). In general, higher content of oleic acid than palmitoleic acid was obtained. In addition, palmitoleic acid content in advanced techniques extracts without pre-treatment was the highest (9.7% to 10.3%). Moreover, no differences in oleic acid content were found between pre-treatment and without pre-treatment extracts, and the lowest oleic acid content was reached by traditional method (12.5%).

In relation to the percentage of PUFA, the highest content was achieved in advanced extraction techniques (39.0% to 39.6%) and the lowest content in traditional techniques (31.7%). The enzymatic pre-treatment did not improve the PUFA extraction (34.1% to 37.7%), but higher PUFA contents were obtained than traditional methods. Other researchers also found a similar fatty acid composition [41,42].

The n-3 PUFAs that were identified in *Isochrysis galbana* were linolenic acid, stearidonic acid, EPA and DHA. Extracts with the highest n-3 PUFA content were produced by UAE and PLE without enzymatic pre-treatment, 33.2% and 31.8%, respectively. While extracts obtained with traditional methods were characterized by a low content of omega-3 fatty acids (25.9%). In general, an enzymatic pre-treatment did not improve the n-3 PUFA content (Celluclast and UAE 28.8%, Viscozyme and UAE 27.8%, Celluclast and PLE 30.2% and Viscozyme and PLE 28.0%) with regards to the original samples. The n-3 PUFA content results of this study were similar to those reported by other authors, where 33.6% n-3 PUFA of total fatty acids was achieved [39].

Linolenic acid content was similar in the different extraction techniques, while stearidonic acid percentage increased for the advanced techniques without pre-treatment UAE and PLE, 12.1% and 12.0%, respectively. EPA content increased in UAE extraction without pre-treatment (3.3%); however, the lowest content was obtained with the Folch method (2.4%), and in the other techniques it remained around 2.7% to 2.9%. DHA content was higher than EPA, the highest content was achieved with UAE and PLE without enzyme pre-treatment (12.0% and 11.4%); a similar content was achieved with advanced techniques with enzyme pre-treatment (10.1% to 10.6%) and the lowest content was reached with the traditional method (9.0%). On the other hand, a low n-6 PUFA (linoleic acid and arachidonic acid) content was found in all techniques, with the lowest value (5.8%) for the Folch method, while the highest content was obtained with PLE methods without pre-treatment (7.2%) and with pre-treatment with Celluclast and PLE (7.5%).

## 3. Materials and Methods

### 3.1. Materials

Commercial dry powder biomass from microalga *Isochrysis galbana* was purchased from Neoalgae Micro Seaweed Products (Gijón, Spain). Chloroform and isopropyl alcohol were purchased from Scharlab S.L. (Sentmenat, Spain) Methanol was purchased from Lab Scan Analytical Sciences (Gliwice, Poland). Hexane and the solvents (2,2,4-trimethyl pentane, methyl tert-butyl ether (MTBE)) used for HPLC analyses were HPLC-grade and purchased from Macron Fine Chemicals (Gliwice, Poland). Absolute ethanol (PRS grade), sodium hydrogen carbonate and potassium hydroxide were purchased from Panreac Quimica S.A (Barcelona, Spain). The water used was Milli-Q grade (Millipore Sigma, Burlington, MA , USA). *Viscozyme*^®®^ from *Aspergillus aculeatus* containing a wide range of carbohydrases, including arabinase, cellulase, beta-glucanase, hemicellulase and xylanase, and *Celluclast*^®®^ containing cellulase from *Trichoderma reesei* were kindly donated by Novozymes (Bagsvaerd, Denmark). Fatty acid methyl esters standard (Supelco 37 FAME Mix) was from Supelco (Bellefonte, PA, USA). Glyceryl trilinoleate, dioleoylglycerol (mixture of 1,3- and 1,2-isomers), 1-oleoyl-rac-glycerol, oleic acid and ethyl linoleate used as HPLC standards was purchased from Sigma-Aldrich (St. Louis, MO, USA). All other reagents and solvents used were of analytical or HPLC grade.

### 3.2. Lipid Extraction of Microalgal Biomass

Lipid extractions from the dry powder microalgal biomass from *Isochrysis galbana* were carried out using different techniques: conventional techniques such as Folch and Bligh and Dyer method; and advanced techniques such as pressurized liquid extraction and ultrasound assisted extraction. The experiments were done at least in duplicate in all cases.

#### 3.2.1. Folch Method

The Folch extraction method was performed following the original procedure described by Folch et al. [17]. Dry powder microalgal biomass (1 g) was extracted with 20 mL of chloroform:methanol (2:1) vortexing for 2 min. The mixture was centrifuged at 3000 rpm for 10 min and the organic layer was collected. The collected organic layers were purified washing with water and centrifuged at 3000 rpm for 10 min. The purified process was carried out three times on the same organic layer. Finally, chloroform layer containing the extracted lipids was collected and evaporated.

The samples were evaporated in a rotary evaporator (Heidolph Hei-Vap Value HB/G3, Germany) under reduced pressure at 40 °C and dried under a nitrogen stream until constant weight. The lipid content was determined gravimetrically and calculated as weight percentage of dry biomass. Lipid extracts obtained were stored in dark vessels with nitrogen atmosphere at 4 °C until their analysis.

#### 3.2.2. Bligh and Dyer Method

The Bligh and Dyer method was done following the original procedure described by Bligh and Dyer et al. [16]. Dry powder microalgal biomass (1 g) was extracted with 15 mL of chloroform:methanol (1:2) vortexing for 30 s. Chloroform (5 mL) was added in the mixture vortexing for 30 s and water (5 mL) was added in the mixture vortexing for 30 s. The blend was filtered with a fold filter. The mixture was centrifuged at 3000 rpm for 10 min and the organic layer was collected. Samples were evaporated and treated as previously described for the other extraction methods.

#### 3.2.3. Ultrasound Assisted Extraction

UAE was carried out with an ultrasound bath S 40H Elmasonic Elma (Singen, Germany) with time automatic control and temperature, and an ultrasound frequency 37 kHz. Dry powder microalgal biomass was weighed (3 g), added in 30 mL mixture solvent hexane:isopropanol (2:1) and introduced in vials of 50 mL. Different times and temperatures were selected for the extraction. Finally, samples were filtered with a fold filter were evaporated and treated as previously described for the other extraction methods.

#### 3.2.4. Pressurized Liquid Extraction

PLE was carried out with an extractor ASE 350 DIONEX (Sunnyvale, California) equipped with stainless steel extraction cells (10 mL volume). Dry powder microalgal biomass was weighed (1 g), mixed with sea sand (ratio 1:10) and loaded into the extraction cell. Then, the extraction cell was filled with the mixture solvent hexane: isopropanol (2:1) used and heated at selected temperature. Static extraction time was 10 min for each experiment and the used solvent volume was 20–25 mL, depending on the temperature and pressure of the cell. Finally, the extract was recovered under a nitrogen stream in vials of 50 mL. Samples were evaporated and treated as previously described for the other extraction methods.

#### 3.2.5. Pre-Treatment with Enzymes

The protocol of the enzymatic pre-treatment of *Isochrysis galbana* biomass with Viscozyme and Celluclast was adapted from the procedure described by Zuorro et al. [43]. The dry microalgal biomass (1 g) and 25 mL of the enzymatic (Viscozyme and Celluclast) solutions were loaded into a thermostatic (50 °C) and magnetically stirred screw-capped glass flasks. The optimal pH 4.8 of the enzymatic solutions was adjusted by adding acetic acid 0.5 N. After different incubation times 2 h, 4 h, 8 h and 24 h, the flask content was centrifuged at 3000 rpm for 10 min.

### 3.3. HPLC-ELSD Analysis

HPLC with Evaporative Light Scattering Detector (HPLC-ELSD) analyses were performed using an Agilent 1260 Infinity HPLC equipped with an Agilent 385 (Palo Alto, CA, USA) ELSD instrument. The chromatographic separation of the different species of lipids (neutral and polar lipids) was performed with a silica normal-phase ACE (250 mm × 4.6 mm i.d. 0.5 µm) column maintained at 30 °C using a ternary gradient as follows: 0–2 min, 99.5% A and 0.5% B; at t = 6.5 min, 70% A and 30% B; at t = 11 min, 63% A, 27% B and 10% C; at t = 18 min, 99.5% A and 0.5% B; and at t = 20 min, 99.5% A and 0.5% B. Eluent A consisted of 2,2,4-trimetilpentane, eluent B consisted of methyl tert-butyl ether and eluent C consisted of 2-propanol. The flow rate was variable (1.0 or 2.0 mL/min). The optimal signal and resolution were achieved with the following ELSD conditions: evaporator temperature = 30 °C; nebulizer temperature = 30 °C; and evaporator gas N2 = 1.6 SLM.

### 3.4. Fatty Acids Composition by GC-MS

Fatty acids composition of all fractions obtained was analyzed by Gas Chromatography-Mass Spectrometry (GC-MS). Previous to the analysis on an Agilent GC–MS series 5975 MSD (Palo Alto, CA, USA), fatty acid methyl esters (FAMEs) were freshly prepared by base-catalyzed methanolysis of the glycerides (KOH in methanol). FAMEs were separated using a HP 88 capillary (100 m × 0.25 mm, i.d. 0.2 µm) (Agilent, CA, USA). 1 µL sample was injected using a split ratio of 1:10. The column was held at 175 °C for 10 min after injection; the temperature was programmed at 3 °C/min to 220 °C and held for 20 min more. Helium was used as gas carrier, at a constant column flow rate of 1.5 mL/min. The injector temperature was 250 °C and the detector temperature was 230 °C. The mass spectrometer was operated at 70 eV with a mass range from 30 to 400 amu. Fatty acids were identified comparing their retention times and the mass spectra (NIST MassSpectral Library Version 2.0) with those obtained from the standards.

## 4. Conclusions

The use of advanced techniques, UAE and PLE, as alternative techniques to the traditional methods to extract lipids from microalgae with n-3 PUFA, proved to be effective due to the increase in performance in short times of the pressurized liquid extraction method. The enzymatic pre-treatment before UAE and PLE allowed fractionating neutral glycerides and polar glycolipids with different omega-3 PUFA composition, which may be used for different aims. The combined method of enzymatic pre-treatment and fast pressurized liquid extraction was very remarkable because it managed to extract more neutral lipids and glycolipids than traditional methods and it also improved the n-3 PUFA content in the fractions, producing bioproducts from microalgae with new potential applications.

## Figures and Tables

**Table 1:** Fatty acid composition (as a percentage of total fatty acids) determined by CG-MS of microalgal lipid fractions at optimal extraction conditions.

% Fatty Acids ^1^								
Fatty Acid	RT (min)	Folch	UAE ^2^	PLE ^3^	Celluclast UAE ^4^	Viscozyme UAE ^5^	Celluclast PLE ^6^	Viscozyme PLE ^7^
14:0	10.4	12.2 ± 0.5	14.1 ± 0.9	13.6 ± 0.2	11.2 ± 0.1	11.8 ± 0.5	12.4 ± 0.3	12.1 ± 0.8
16:0	12.9	21.5 ± 1.1	15.4 ± 0.2	15.5 ± 0.9	17.2 ± 1.4	18.7 ± 1.1	17.4 ± 1.5	17.4 ± 1.2
16:1	14.0	7.7 ± 0.2	10.3 ± 0.3	9.7 ± 0.1	7.2 ± 0.8	7.6 ± 0.6	7.6 ± 0.1	7.3 ± 0.9
17:0	14.5	1.6 ± 0.5	1.9 ± 0.3	2.2 ± 0.1	2.6 ± 0.1	2.7 ± 0.1	2.3 ± 0.1	2.9 ± 0.1
18:0	16.3	8.7 ± 1.6	-	-	6.1 ± 1.8	5.7 ± 1.2	2.0 ± 0.8	2.6 ± 0.4
18:1	17.5	12.5 ± 0.6	14.2 ± 0.1	14.1 ± 0.3	13.9 ± 0.1	13.7 ± 0.5	15.8 ± 0.7	15.3 ± 0.5
18:2	19.2	2.9 ± 0.3	3.2 ± 0.4	3.3 ± 0.3	3.3 ± 0.1	2.8 ± 0.1	3.5 ± 0.2	3.1 ± 0.3
18:3	21.2	4.5 ± 0.4	5.8 ± 0.2	5.5 ± 0.1	5.1 ± 0.0	4.6 ± 0.3	6.1 ± 0.2	5.6 ± 0.2
18:4	22.5	10.0 ± 0.8	12.1 ± 0.2	12.0 ± 0.6	10.6 ± 0.5	9.8 ± 0.6	11.1 ± 0.5	10.7 ± 0.3
20:4	24.0	2.9 ± 0.7	3.2 ± 0.1	4.0 ± 0.2	3.7 ± 0.3	3.6 ± 0.3	4.0 ± 0.4	4.0 ± 0.2
20:5	27.5	2.4 ± 0.3	3.3 ± 0.5	2.9 ± 0.0	2.9 ± 0.1	2.7 ± 0.4	2.9 ± 0.1	2.8 ± 0.3
n.i	28.5	1.5 ± 0.3	1.6 ± 1.1	2.2 ± 0.2	2.6 ± 0.3	2.4 ± 0.3	2.0 ± 0.0	2.0 ± 0.0
n.i	30.9	2.5 ± 0.1	2.9 ± 0.7	3.5 ± 0.2	3.4 ± 0.5	3.1 ± 0.6	2.7 ± 0.2	4.1 ± 1.6
22:6	33.7	9.0 ± 1.3	12.0 ± 0.3	11.4 ± 0.6	10.2 ± 0.6	10.6 ± 1.1	10.2 ± 0.1	10.1 ± 0.9
SFA		44.1	31.4	31.4	37.1	39.0	34.1	35.0
MUFA		20.2	24.6	23.8	21.1	21.4	23.4	22.6
PUFA		31.7	39.6	39.0	35.8	34.1	37.7	36.3
n-6		5.8	6.4	7.2	7.0	6.3	7.5	7.1
n-3		25.9	33.2	31.8	28.8	27.8	30.2	28.0
n-6/n-3 Ratio		0.2	0.2	0.2	0.2	0.2	0.2	0.2

RT, retention time; UAE, ultrasound-assisted extraction; PLE, pressurized liquids extraction; SFA, saturated fatty acids; MUFA, monounsaturated fatty acids; PUFA, polyunsaturated fatty acids. ^1^ Results expressed as percent over the total content (relative content). Values are the mean ± SD of two determinations; ^2^ UAE 50 °C 15 min; ^3^ PLE 100 °C; ^4^ Celluclast pre-treatment 2 h and UAE 50 °C 15 min; ^5^ Viscozyme pre-treatment 2 h and UAE 50 °C 15 min; ^6^ Celluclast pre-treatment 2 h and PLE 100 °C; ^7^ Viscozyme pre-treatment 2 h and PLE 100 °C.
